# Federated learning: Overview, strategies, applications, tools and future directions

**DOI:** 10.1016/j.heliyon.2024.e38137

**Published:** 2024-09-20

**Authors:** Betul Yurdem, Murat Kuzlu, Mehmet Kemal Gullu, Ferhat Ozgur Catak, Maliha Tabassum

**Affiliations:** a Department of Electrical and Electronics Engineering, Izmir Bakircay University, Izmir, Turkey; b Batten College of Engineering and Technology, Old Dominion University, Norfolk, VA, USA; c Department of Electrical Engineering and Computer Science, University of Stavanger, Rogaland, Norway

**Keywords:** Data privacy, Federated learning, Distributed machine learning

## Abstract

Federated learning (FL) is a distributed machine learning process, which allows multiple nodes to work together to train a shared model without exchanging raw data. It offers several key advantages, such as data privacy, security, efficiency, and scalability, by keeping data local and only exchanging model updates through the communication network. This review paper provides a comprehensive overview of federated learning, including its principles, strategies, applications, and tools along with opportunities, challenges, and future research directions. The findings of this paper emphasize that federated learning strategies can significantly help overcome privacy and confidentiality concerns, particularly for high-risk applications.

## Highlights

• In-depth Review and Categorization of Federated Learning Strategies.
• Examination of Security Concerns in Federated Learning.
• Applications of Federated Learning Across Various Domains.
• Insight into Federated Learning-based Frameworks and Tools.
• Future Directions and Emerging Opportunities in Federated Learning.

## 1 Introduction

In recent years, Artificial Intelligence (AI) has become more popular with advanced computing technologies and has been applied in almost all sectors. For instance, in the gaming industry, when AlphaGo [1] defeated the top human Go players, it demonstrated the huge potential of AI. More complex cutting-edge AI technology was seen in many sectors such as healthcare, finance, and many applications. With the success of AlphaGo, a new technology called Federated Learning (FL) came into AI, providing the solution to the problems faced earlier. Federated learning is a distributed machine learning (ML) technique that uses multiple servers to share model updates without exchanging raw data. This helps to overcome the sensitivity to data and privacy-protected technology [2].

FL has been proposed as an alternative approach under the coordination of a central server, and a global model is trained in the form of a federation of devices that participate [3], [4], [5]. However, in terms of data science, there are some drawbacks.

First, AI and ML models require many datasets to train them successfully. In recent years, datasets have grown larger, and models have become more complex. Training ML models require optimization and distribution of model parameters on multiple machines [4]. Especially with medical data, the main problem arises when a model is trained with a huge dataset. It becomes more critical when working with medical data as the information becomes highly sensitive. There is great concern among the public, media, and government about recent news of leaks. For example, Facebook's recent breach of privacy caused several protests about data privacy [6]. In response, the European Union, on 25 May 2018, enforced the General Data Protection Regulation (GDPR) [7] to strengthen laws to protect data security and privacy. European Parliament also regulates that users have complete power over their data [8]. People are also paying more attention to their data and where these data are used these days [9], [10]. Thus, prior consent is required to use such data.

Second, if the number of datasets is limited, there is a restriction to the development of the training model. That is, the relation between the number of datasets and the model training is proportional. In a dataset, there is the possibility of the presence of all kinds of scenarios. Such as datasets that help detect a chronic wound to make it possible to diagnose the disease before any severe measures. However, obtaining this kind of data is difficult due to its sensitivity and high regulation [11].

Third, when dealing with healthcare data, a person's data becomes highly sensitive. This type of data contains private information. Moreover, while a large dataset is needed to train an AI model, the limited number of datasets hinders the development of ML models [12]. Due to its sensitivity and different types of regulations, obtaining this kind of data is difficult [13].

For example, the initial version of AlphaGo relied on 160,000 sets of human chess data. This had the ability to defeat beginner-level players. On the contrary, AlphaZero used human-generated chess data that has been shown to be capable of defeating professional players [14]. FL-based training approaches for a neural network that includes image classification [15] or natural language process [16] are promising along with new methods developed daily, and several new mobile applications are being developed. For example, the prediction of Google keyboard words was improved using FL by Hard et al. [17].

### 1.1 Research methodology

The research process for this review paper focused on a systematic way to provide an extensive survey of the overview, strategies, applications, tools, and future directions of Federated Learning. For this, publications and preprints consisting of review articles, research articles, book chapters, and conference papers on academic databases consisting of Web of Science, Elsevier, IEEE Xplore, Scopus, Springer, and Google Scholar were examined.

During the research studies, the main keyword used in searches was “federated learning”. Additionally, keywords involved in data privacy and security, data heterogeneity, model aggregation, framework, and application topics were used in the review. Since it is a timely and wide topic, there may be unobserved publications on this subject with the possibility that similar terms may be overlooked.

In this research, since 2016 was the first year of proposing the FL concept [4], the publications after this date were examined. Table 1 shows the number of selected articles found with the given main and additional keywords, the selection conditions of which were previously specified.

**Table 1:** Total number of selected articles relevant to the topic.

Keywords	Total number
“Federated learning”	3768
“Application” + “Federated learning”	3531
“Framework” + “Federated learning”	3304
“Privacy” + “Federated learning”	2956
“Strategies” + “Federated learning”	2919
“Security” + “Federated learning”	2543
“Tool” + “Federated learning”	2343
“Overview” + “Federated learning”	1918
“Aggregation” + “Federated learning”	1865
“Attack” + “Federated learning”	1832
“Heterogeneity” + “Federated learning”	1203
“Blockchain” + “Federated learning”	1254

As can be seen from the table, although it is a new field of study, there are many studies in almost every sub-branch of this subject, and in this paper, these branches are explained. Overall, this paper is organized as follows. Section 2 provides an overview of Federated Learning, divided into two subsections covering the characteristics and categorization of FL. Section 3 provides various FL strategies. The security concerns associated with FL are addressed in Section 4. Section 5 presents various applications of FL. Section 6 examines some of the FL frameworks and tools. In the last section, opportunities, challenges, and future directions in FL are explained.

## 2 Overview of federated learning

Federated learning aims to build global AI models for multiple parties with diverse interests. Instead of collecting and combining data from different locations and gathering them in a central location, FL processes data from the place where it ensures the security of sensitive data and models.

Federated learning has undergone significant development since its introduction. Initially, researchers proposed distributed stochastic gradient descent (SGD) as a way to train deep neural networks on multiple devices. However, this approach requires that each device transmit its local model to a central server after every iteration, raising privacy and communication issues. In response, Google researchers proposed the FL approach in 2016 [18], which allowed model training to occur on the devices themselves without requiring the transmission of raw data to a central server. This approach has been extended along with various federated learning architectures and algorithms. An extension is the use of differential privacy introduced by Google researchers [19], which is a mathematical framework to quantify and guarantee privacy in the AI/ML concept. Random noise is added to the model updates generated by participating devices in differential privacy. This approach has also been extended by using heterogeneous data [6], where devices may have different data distributions. In addition, federated learning has been extended to other domains, such as federated optimization [20] and federated reinforcement learning (FRL) [21]. In federated optimization, multiple entities cooperate to optimize a shared objective function, while agents learn to act in a decentralized environment without central coordination in FRL. These extensions will significantly contribute to the potential applications and open new research directions in federated learning. The future of federated learning promises additional developments, including the exploration of federated learning with non-IID data, the integration of federated learning with blockchain technology, and the development of more robust and scalable federated learning algorithms.

In recent years, federated learning has become more mature with its characteristics and categorizations depending on how the data is distributed, stored, and interrelated. The characteristics and categorization of FL are discussed in the following subsections.

### 2.1 Characteristics of federated learning

FL is a field of distributed machine learning, which puts a great deal of effort into data privacy. The latest studies also pay attention to distributed systems that preserve privacy. It connects multiple nodes located in different locations but is connected via a communication network. The network is under the control of the central server, and each node undertakes different parts of the same task to complete it. The main characteristics of FL are discussed below.

• **Universal cross-organization scenario:** FL was originally proposed by Google as a distributed ML technology. This allows participants to build a global model while keeping their underlying data local. The initial concept of FL is expanded to encompass all decentralized ML techniques that preserve privacy [6].
• **Massively non-identically independent distribution:** In FL, data is massively widespread among a huge number of edge notes or devices. The data available at each node might be less than the total number of nodes.
• **Decentralization:** It is not completely used in a technical sense. However, there is no definitive center. Each client is not determined to be the center, but they influence the central model. Parameter servers are used as a central server that is dominant enough to distribute the data. It works as a resource to obtain efficient collaboration [22].
• **Equality of status for each node:** In FL, all parties receive the same facility. This means that who has the largest number of data has the largest mass.

### 2.2 Categorization of federated learning

Federated Learning can be categorized in different ways based on data distribution. In this paper, the two most popular categorizations are explained, i.e., vertical federated learning and horizontal federated learning.

**Figure 1:**
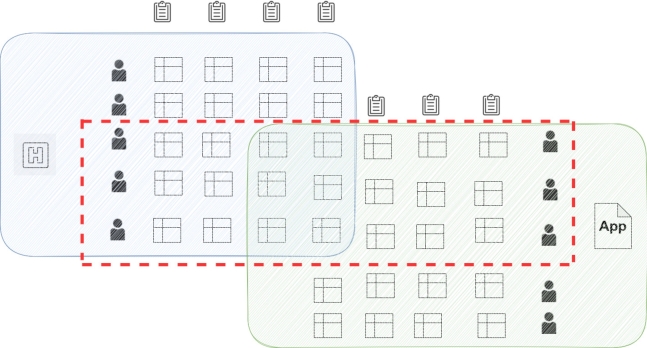
An example of vertical FL-based application [2].
• **Horizontal Federated Learning:** In the case of horizontal FL, similarity exists in the data features that are spread across various nodes. Meanwhile, those data are different in sample space. The existing FL algorithms are primarily aimed at various applications of IoT devices or smart devices [2]. In these scenarios, FL is classified as horizontal-federated learning. Here, data in the sample space may differ drastically but have similar features. Furthermore, a hierarchical heterogeneous horizontal FL frame was introduced to meet the criteria for limited labeled entities [25]. Adapting a heterogeneous domain can solve the issue of label shortage adopted multiple times using each participant as the target domain. In real applications, such as healthcare, data collection is inseparable from a huge number of work. It is almost impossible for each hospital to build a data pool to share data. Therefore, in horizontal FL, multiple parties train a model with similar datasets from different sources [26]. An example of this is illustrated in Fig. 2.

• **Vertical Federated Learning:** It is suitable in cases where data is partitioned in the verticle direction in accordance with feature dimension. Vertical federated learning (VFL) is where data features are split among multiple parties. It is the concept that collaborates to train a model on a dataset. Here, all parties have homogeneous data. That means that they overlap partially on sample ID but are different in feature space.
  For example, a medical organization intends to work to identify and predict diseases related to diabetes. According to research, people with high blood pressure and obesity are likely to develop Type-2 diabetes [23]. Therefore, analyzing the weight and age of patients can give rough dimensions along with their medical history. However, a young patient who consumes more sugar and lacks physical activity but is not obese or has high blood pressure is more prone to suffer from diabetes. This diagnosis is not predicted and is personalized due to lack of information. With the development of FL, some companies that work with smartphone application datasets, such as step counters or dietary structures, can cooperate with each other. This can be done without sharing the raw data transmissions as the figure shows. Normally, taking out similar entities that possess different characteristics to get joint training is a path taken by scholars.
  Another example is that patient data can be present in different healthcare organizations. As the identity of the patient is secret, those two different data cannot be merged to train a model. In order to address this issue, it is necessary to train a machine learning model through a collaborative effort that will be conducted within the appropriate premises. Unlike horizontal FL, vertical FL poses additional challenges in terms of entity resolution [24]. Unlike the straightforward approach of aggregating all datasets on a common server, this method is not effective in vertical FL. An example of how vertical FL works is given in Fig. 1. It can be inferred that there is still more potential for enhancing vertical FL to be utilized in a more intricate ML approach.

**Figure 2:**
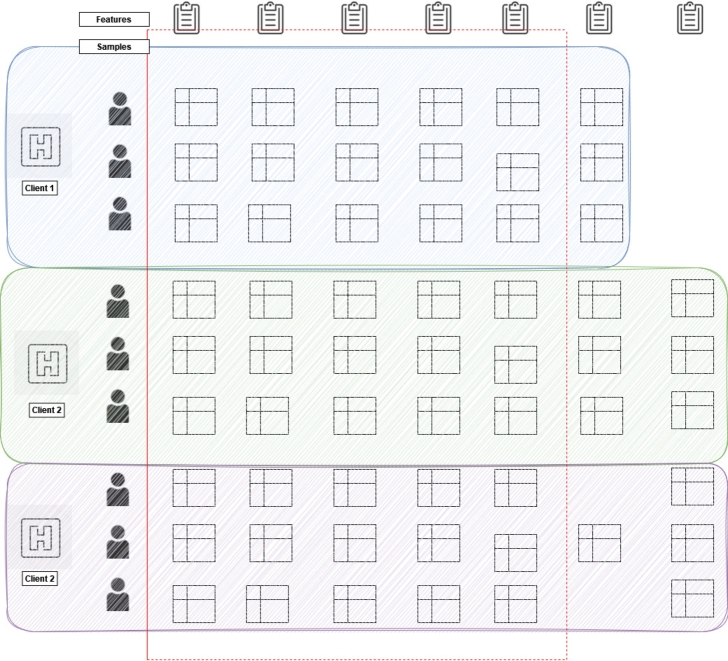
An example of horizontal FL-based application [2].

In FL models, one model can have multiple characteristics discussed in the previous section. The different types vary in their needs. The categorization is not mutually exclusive. In Table 2, the comparison of these two models is shown according to their feature and sample diversity, training point, data sharing, privacy and security, scalability, computation features, and communication resources.

**Table 2:** Comparison of categorization of federated learning [27].

Criterions	VFL	HFL
Features	Different	Common
Samples	Common	Different
Training	Locally on client devices	
Shared with Server	Local model updates	
Get from server	Global model updates	
Data sharing with global server	No	
Privacy and security	Data is not shared with the global server	
Scalability	Low	High
Computing power	Bounded with the device capacities of clients	
Communication resources	Consumes less than HFL	Consumes more than VFL
Pros	Huge and detailed datasets	**Table:** Lower communication cost than centralized ML
Cons	**Table:** Works with a small number of clients	Data heterogeneity

## 3 Federated learning strategies

In FL, multiple parties can collaborate without sharing raw data. Each party shares model updates with a central server. The data go through aggregation by the central server and are sent to local sources. In this way, the model is trained while benefiting all parties by providing privacy.

Distributing the data across different devices is more convenient than gathering all the data in one location or device. To achieve this, a variety of federated learning strategies have been developed [28]. Selected ones from the commonly used ones are explained in the next subsections.

### 3.1 Algorithms

#### 3.1.1 Federated averaging

Federated Averaging, also known as FedAvg, is the most popular FL strategy. In FedAvg, a local model gets trained by each client with their own data. Allocating parameters to a large number of clients, recycling, training, and updating them are done by the central server while not sharing the client's information. The whole communication is done by the central client. The model parameters across all parties are averaged by aggregation. After that, each client receives the global model, and this process is then continued until the model reaches convergence. This process is explained in Appendix A - Algorithm 1. This model can provide the privacy promised by FL models. The key factor behind this is to minimize communication overhead. In the ML model, this is a promising approach. There are some significant advantages of FedAvg over other centralized ML models:

• It provides data privacy. Data is stored in the local model while the model updates are shared. In this case, a central server is not used to share data.
• Model updates are only shared between participants. It results in a significant amount of communication overhead, which makes it environmentally friendly.
• It is scalable to large-scale FL scenarios with a large number of participants. The participants have the freedom to join or leave the network at any time.

#### 3.1.2 FedAdam

FedAdam is the reformulation of the Adam optimizer for FL. In this approach, training data is distributed among devices. The moving average of the model update follows the momentum decay rule. This technique adaptively updates the model's weights by utilizing moving averages and adjusting the learning rate for each weight. The exponential moving average of the first moment of the gradient is computed. The weights are updated with a learning rate and adapted based on the estimated variance and mean gradient. The primary distinction between FedAdam and the standard Adam algorithm is the use of a federated average to calculate the gradient across all devices. The model can also run well with the new data while preserving the old data.

#### 3.1.3 FedYogi

In the case of FL, this strategy lets users train a model where the identity of the clients is not shared with the central server. This process avoids the collection of sensitive data that can breach privacy. Mostly, this is achieved by learning a single global model. This model is for all users, even if they are using different data distributions. For instance, users of a device prefer different suggestions while training a model. The personalization of this heterogeneity is the motivation behind each user's global model. In the case of privacy, learning the full model is prohibited for privacy reasons. That is why Federated Optimization is adapted with Yogi.

#### 3.1.4 FedAdagrad

FedAdagrad is the short form of Federated Adaptive Gradients, which is an extension of the gradient descent optimization algorithm. The gradients seen for the variable, i.e., partial derivatives, are optimized and observed throughout the search. Regarding all the model parameters, the algorithm is based on adapting the learning rate and first-order information with some properties of second-order methods and annealing. With the growth of the dynamic rate, it is inversely proportional to the magnitude of the gradient. The relationship between large gradients and smaller learning rates is proportional. The scale of gradience varies in each layer by several orders. Moreover, the denominator of the scaling coefficient has a similar effect as annealing, which gradually decreases the learning rate over time. The Appendix A - Algorithm 2 describes all FedAdam, FedYogi, and FedAdagrad strategies.

#### 3.1.5 FedProx

This strategy is used to lessen statistical heterogeneity. It introduces terms that regularize each local training loss and is built on FedAvg [29]. The local model and its deviation are also controlled by this model. Re-parameterization does some minor modifications but it is a better version of FedAvg. It is important for both theoretical and practical fields. In terms of practical field, robust convergence is demonstrated compared to FedAvg [30]. Meanwhile, in theory, in device-level systems, convergence is provided that allows each device. Between FedProx and FedAvg, the first one is more stable and provides accurate convergence. The FedProx algorithm is represented in Appendix A - Algorithm 3.

#### 3.1.6 Scaffold

Stochastic Controlled Averaging for Federated Learning is also known as Scaffold. The training process is coordinated across different nodes across the network as expected. The Scaffold algorithm is given in Appendix A - Algorithm 4. This approach reduces the need for multiple rounds of communication and resolves the issue of heterogeneity [31]. Furthermore, a server-side learning rate is employed to regulate variates and reduce control variates. Ultimately, it allows for collaboration between multiple devices with different data sources.

### 3.2 Comparison of the FL strategies

In this section, the applications given in the literature are examined and a comparative evaluation of the FL strategies used is accomplished.

In a study by Gao et al. [32], in addition to the FL models trained with the previously mentioned known strategies such as FedAvg, FedProx, and Scaffold, the model trained with the FedDC strategy they proposed was also compared. Thus, the effects of these algorithms for a more convergent model due to heterogeneous datasets were investigated. As a result of studies conducted with different datasets, the FedAvg method had the most round numbers to converge to the target accuracy. Although a higher or approximate number of rounds were obtained with FedProx, trainings were completed faster than FedAvg in some cases. With Scaffold, fewer and faster training processes were completed compared to both methods, and the same percentage of accuracy was achieved with all of them. The FedDC strategy, a combination of FedProx and Scaffold, showed much better results than others for both IID and non-IID datasets. In the final comparison results, they determined that FedDC is robust and effective in full and partial client participation [32].

Nguyen et al. conducted a study with the CIFAR-10 dataset using strategies such as FedAvg, FedProx, Scaffold, FedNova, FedAdam, FedAdagrad, and FedYogi and their combinations [33]. In the obtained test accuracy graph, it was observed that the best results were achieved with the ProxYogi strategy, which is the combination of FedProx and FedYogi [33]. This study was also conducted with a non-IID dataset.

In another study, training was carried out with medical data [34]. FedAvg, FedAdam, FedYogi, FedAdagrad, FedProx, and FedAvgM strategies were used to predict in-hospital mortality and acute kidney injury. In the results, it was given that the training with FedProx had the lowest accuracy for the prediction of acute kidney injury. Nearly all of them obtained highly accurate training results for predicting hospital mortality, but FedAdam had a slightly lower result. They also highlighted that the basic FL algorithms FedAvg and FedAvgM are the best for machine learning tasks, and FedAvg performs well with the IID datasets [34].

As can be understood from the literature, there are cases where the use of each FL strategy is advantageous. An overview of these advantages for commonly used strategies is given in Table 3.

**Table 3:** Comparison of FL strategies.

FL Strategy	Utility
FedAvg	Simple and common
FedAdam	Effective optimization
FedYogi	More stable learning
FedAdagrad	Automatic learning rates
FedProx	Better for non-IID data
Scaffold	More stable convergence

## 4 Security concerns for federated learning

FL is a distributed machine learning approach in the AI/ML concept that involves multiple parties, which introduces various security concerns regarding the privacy, confidentiality and integrity of the data used in the collaborative learning process. In this section, the main security concerns associated with federated learning are discussed along with potential solutions to mitigate these risks.

### 4.1 Data privacy

To mitigate the risk of data privacy, several privacy-preserving techniques have been proposed. One of these methods is differential privacy [35], which adds random noise to the updates sent by each device to obfuscate individual contributions. For example, let *X* be the input data from a device and f(X) be the corresponding model update. Then, the noise added to f(X) is drawn from a probability distribution $N(0,\sigma^{2})$, where *σ* is the privacy parameter that controls the level of noise added. Mathematically, the noisy update can be expressed as $f^{\prime }(X)=f(X)+\eta$, where $\eta ∼N(0,\sigma^{2})$. The privacy guarantee of differential privacy can be quantified using the concept of *ϵ*-differential privacy, which limits the amount of information that can be inferred about any individual device's data from the overall output of the federated learning algorithm. Specifically, a federated learning algorithm is said to be *ϵ*-differentially private if, for any two input datasets *X* and $X^{\prime }$ that differ by a single record, and for any output *S* of the algorithm, the following condition holds:

(1) $$\frac{Pr[S(X)]}{Pr[S(X^{\prime })]}\leq e^{\epsilon }$$

where Pr[S(X)] and $Pr[S(X^{\prime })]$ denote the probability of obtaining the output *S* on datasets *X* and $X^{\prime }$, respectively.

Another method is secure multi-party computation (SMC) [36], which enables collaborative computation on encrypted data without revealing the raw inputs. For example, it is assumed that $X_{1},X_{2},...,X_{n}$ present the private inputs of *n* devices participating in the federated learning process. In SMC, first, each device encrypts its input utilizing a public key, i.e., generates the encrypted data, and then sends the data to a central server. The central server is responsible for computation on the encrypted data as well as returning the encrypted result to the devices to decrypt the data using the private key.

The security of SMC is based on cryptographic techniques, such as homomorphic encryption (HE), which allows computations on encrypted data. It is assumed that Enc(X) is the encrypted version of input *X*, Dec(X) is the decryption function, and let *f* be a function operating on encrypted data. HE ensures the function can be performed on encrypted data without revealing the underlying inputs, i.e., $Dec(f(Enc(X_{1}),Enc(X_{2}),...,Enc(X_{n})))\approx Enc(f(X_{1},X_{2},...,X_{n}))$.

### 4.2 Model poisoning attacks

FL includes model updates from multiple devices to form a global model. However, FL can be exploited by malicious participants, i.e., injecting poisoned updates to corrupt the global model or model poisoning attacks. This can lead to incorrect predictions or leakage of sensitive information. Several defense mechanisms have been proposed for model poisoning attacks.

One mechanism is anomaly detection methods based on statistical methods (clustering or outlier detection) or AI/ML (such as neural networks or decision trees). The anomaly detection method is used to identify and remove malicious updates. This can be explained mathematically as follows:

• Given $U_{t}=u_{1},u_{2},...,u_{n}$: the set of updates received at time *t*, and $f_{t}$: the global model at time *t*.
• Identify updates $u_{i}\in U_{t}$, which do not align with the current global model $f_{t}$, i.e., $||f_{t}-u_{i}||>\theta$, where *θ* is a predefined threshold.
• Define the distance metric, i.e., $\left||f_{t}-u_{i}|\right|$, by using the Euclidean distance or the cosine similarity.
• Identify and remove malicious updates if needed.

An alternative mechanism is robust aggregation algorithms, which can be defined as an optimization problem solved using various techniques, such as gradient descent or stochastic optimization. The robust aggregation algorithms aim to reduce the effect of malicious updates on the global model. This can be simply expressed mathematically as follows:

• Given $f_{t}$: the global model at time *t*, and $U_{t}^{m}=u_{1}^{m},u_{2}^{m},...,u_{n_{m}}^{m}$: the set of malicious device updates at time *t*, $L(f_{t},U_{t})$: the loss function of the global model in the set of updates $U_{t}$, and *k*: a constraint that limits the maximum number of compromised updates.
• Define the optimization problem as follows:
  (2) $$\min_{f_{t}}E_{U_{t}﹨U_{t}^{m}}[L(f_{t},U_{t})]\;\text{subject to}\;|U_{t}^{m}|\leq k$$
• Apply the defined optimization algorithm to the global model.

### 4.3 Model inversion and membership inference

FL can be vulnerable to model inversion and membership inference attacks. Model inversion attempts to uncover sensitive information from the trained model, while membership inference attempts to determine if a particular data point is used in the training. These attacks can be more critical particularly high-risk applications, such as healthcare or finance.

Fortunately, a variety of countermeasures have been developed to enhance the security of federated learning against these attacks, which include model regularization, adversarial training, and gradient obfuscation. Each countermeasure method is briefly explained below.

• **Model regularization** is an additional regularization term to the loss function during training, which penalizes the model for learning sensitive information. This can be given mathematically as:
  (3) $$L_{\text{regularized}}=L+\lambda \cdot \text{R}(f)$$
  where L is the original loss function, *λ* is a regularization parameter, and R(f) is a regularization term to encourage the model to forget sensitive information.
• **Adversarial training** is the training process against adversarial examples generated specifically to extract sensitive information. Mathematically, this can be given as a min-max optimization problem:
  (4) $$\min_{f}\max_{x^{\prime }\in X^{\prime }}L(f,x,x^{\prime })$$
  where *x* is the original input, $x^{\prime }$ is the adversarial example, and $L(f,x,x^{\prime })$ is the loss function that captures the vulnerability of the model to model inversion attacks.
• **Gradient obfuscation** protects sensitive information by perturbing gradients during the training process, making it more difficult for an adversary to perform model inversion attacks.

Additionally, a variety of countermeasures such as privacy-preserving data synthesis and differentially private aggregation can also be utilized for membership inference attacks. Each countermeasure method is briefly explained below.

• **Privacy-preserving data synthesis** refers to the process of generating synthetic data that maintains the statistical characteristics of the original data while ensuring the confidentiality of individual data points. It uses techniques such as generative adversarial networks (GANs) or differential privacy mechanisms. Mathematically, this can be given as:
  (5) $$\overset{ˆ}{X}=\text{Synthesize}(X)$$
  where *X* is the original dataset and $\overset{ˆ}{X}$ is the synthesized dataset. The synthesized dataset $\overset{ˆ}{X}$ can be used for training the federated model while protecting the privacy of individual data points.
• **Differentially private aggregation** is to protect membership privacy by adding random noise to the aggregation process. Mathematically, this can be given as:
  (6) $$\text{Agg}(U_{1},U_{2},...,U_{n})=\frac{1}{n}\sum_{i=1}^{n}U_{i}+\text{noise}$$
  where $U_{1},U_{2},...,U_{n}$ are the updates from the participating devices, and noise is a random noise term that ensures differential privacy guarantees.

These techniques provide additional security measures to mitigate the risks associated with model inversion and membership inference attacks in federated learning.

## 5 Federated learning-based applications

FL can be used in different fields, and any field that deals with a person's private information can benefit from FL. Below are some of the applications that can use and benefit from FL models.

### 5.1 Healthcare

FL shares its data without revealing the identity of the users. Each medical institute might have some data about their patient. Still, the amount of data lacks sufficiency to train a model [37]. This makes the use of FL in this field very attractive. Some of the applications in healthcare are discussed as follows:

• **Electronic Health Records (EMR):** It has a lot of clinical components, the authors [38] suggested an attempt to use tensor factorization models. It would do phenotype analysis and obtain concealed medical data of patients. It is the first attempt at FL learning. Their proposed graph-based attention model was used in disease diagnosis prediction with the records of adult patients and intensive care unit patients, and heart failure prediction studies. The model they proposed as a result of studies conducted with the data of patients between the ages of 20-40 in intensive care disease prediction ensured them to achieve 10% higher accuracy than training with RNN [38]. The authors [39] explored EMR differently while using Federated Settings. It was used to predict the mortality rate of cardiac patients by using EMRs from different hospitals.
• **Biomedical Image Analysis:** FL has been used in this sector. For example, Silva et al. [40] put forward FPCA (Federated principal components analysis). It extracted features from MRI that come from different medical centers.
• **EEG Classification:** A Hierarchical Heterogeneous Horizontal FL (HHHFL) was proposed by Gao et al. [25]. They overcame the challenge of limited labeled samples along with the provision of privacy [2].
• **Clinical Prediction:** To predict disease, beforehand FL can be used. As described by the authors Pfohl et al. [39], FL establishes efficacy over centralized and local data. It also performs FL differently.

Overall, FL can be used to provide effective ways in healthcare that will help to make life easier.

### 5.2 Internet of Things (IoT)

**Figure 3:**
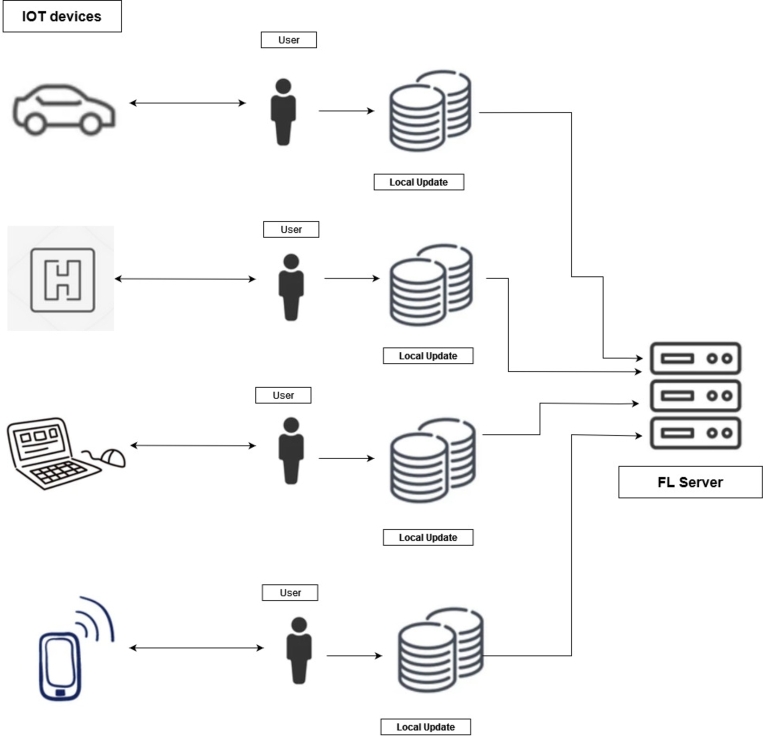
Applications of federated learning with different IoT devices.

Federated learning has several potential applications in the IoT industry. Fig. 3 shows various applications of FL and how different IoT devices take part in training an FL model. Here are some of the potential applications of federated learning in IoT:

• **Smart Transportation:** Intelligent Transportation System (ITS) has been implemented by using AI/ML for the past few years. It is done using centralized vehicular data learned from the data center [41]. This requires sharing data in an untrusted environment, which is a safety issue for FL and is used to provide privacy and safety [42].
• **Smart City:** The use of AI has been increasing over the years. The creation of a smart city that can enhance the quality of life for citizens living in urban areas is needed. By supplying seamless delivery of food, water, and other necessary items to their users [43]. To create a smart city, many sensors are needed. Also, AI and ML have the ability to take care of huge numbers of big data that are usually generated from sensors [44]. FL offers a more attractive and effective way by enabling decentralized methods. For example, an environmental monitoring framework based on fog computing was proposed in study [45]. The studied multi-source heterogeneous dataset was collected from the IoT sensors in Beijing.
• **Smart Grid:** Smart grid is an important factor in terms of increasing industrial systems as it provides the necessary power to the energy sources of the household and industry [46]. Smart grid operations can be helped by FL with intelligent solutions. For example, FL can be used to create a federated predictive power scheme that can run a recent neural network locally. That can estimate the amount of power demands by inspecting the history of a customer's usage. This can help construct a global prediction model [47]. In the research [47], a power consumption prediction model for GPU-based edge data centers was constructed. Their model is based on ANN and achieves a normalized root mean square deviation error of less than 7.4% compared to actual measurements.

The FL model can help in the IoT sector in different ways. The use of different technological products is increasing day by day, and this requires privacy, too. FL can provide this by leveraging IoT products.

### 5.3 Internet of Underwater Things (IoUT)

IoUT stands for Internet of Underwater Things and has garnered rapid momentum recently [48]. The use of FL in IoT networks has been expanding rapidly in recent years. It spans applications on defense, environmental monitoring, exploration of the sea, etc. Like any other FL system, IoUT also uses a traditional ML model. Below, the various applications of IoUT are discussed.

• **Environmental Monitoring:** The environment consists of different elements like water, air, soil, etc. In terms of them, water is an essential one, and it is used in everyday life. The quality of the water always needs to be monitored because contaminated water can cause some serious problems. FL can be used to monitor the quality of water at all times. So that, no man-made pollution happens, like contaminating the water due to oil spills.
• **Underwater Exploration:** This helps to discover lost treasures such as underwater ruins of civilization and other natural resources. The underwater environment is used to experiment with some new technology. How these technologies are affected is also investigated using several sensors. H. Zhao et al. [49] proposed to use a “federated meta-learning enhanced acoustic radio cooperative framework”, called as ARC/FML, for data collecting from distributed sources. This stated technique helps in sharing sensitive data regarding underwater exploration. DeepSink and RF Channel were used to conduct the experiment. The proposed model provided an accuracy of 97%.
• **Disaster Prevention and Mitigation:** The underwater is always susceptible to various disasters. They can be both natural and man-made. Disasters that are man-made are mostly oil spills, and poisonous chemical spills which mostly occur due to underwater experiments. On the other hand, natural disasters are tsunamis, earthquakes, and underwater volcano eruptions. The Deepwater Horizon incident in 2010 [50] is one of the worst man-made disasters to be recorded. It occurred due to the leakage of a significant amount of natural gas, which resulted in the gas rising and catching fire [51]. On the other hand, the most horrific natural disaster occurred in 2004, which was the earthquake and tsunami in the Indian Ocean [51]. FL-enabled IoUT solutions can significantly provide help in preventing these disasters by sending the data in real-time.
• **Defenced Military:** Defensive naval operations traditionally involve human-operated underwater vessels to carry out tasks such as detecting submarines, mine warfare, recovery operations, and surveillance. However, advancements in technology have led to the development of Underwater Wireless Sensors, or UWSN, which are a type of wireless sensor network that allows for underwater activities to be carried out without human intervention. UWSN are capable of detecting and classifying objects in the underwater environment. The US Navy places great emphasis on real-time data sharing between ships, submarines, drones, and intelligence analysts onshore [52]. FL is used for this purpose, too.

These are some of the applications of FL in terms of IoUT. There are many more ways that FL can be used.

### 5.4 Industrial engineering

FL has several application sectors in different industries, and some of them are mentioned below.

• **6G Industry:** In the recent growth in data traffic, ML has garnered a huge amount of attention. In the development of the sixth generation (6G), it has become vital [53].
• **Image Detection:** FL can also be applied to perform visual inspection task [54]. It can be used to help detect defective products in the manufacturing industry.
• **Image Representation:** In image field, Liu et al. [55] describes about how vision-and-language as a flashpoint. They also discuss how FL can diversify these tasks for better grounding.
• **Unmanned Aerial Vehicles:** The work of unmanned aerial vehicles by using FL is given by Mowla et al. [56]. It was discussed how these vehicles can detect malicious attacks by using FL.
• **Electrical Vehicles:** Electric vehicles are getting popularized now. An FL is designed by Saputra et al. [57] that can predict the vehicles' energy demand and methods of efficient charging stations. This can prevent energy congestion in transmissions.
• **Financial Field:** The use of credit cards and transactions in various fields is increasing day by day. Yang et al. [58] leveraged FL to detect credit card fraud efficiently. This is a significant contribution to the field of finance.
• **Text Mining:** An industrial grade federated framework was used by Wang et al. [59] on Latent Dirichlet Allocation. It is used to assess real data for spam filtering.

### 5.5 Integrating FL with technologies

FL is used in studies in different fields and with different data, as well as in integration with different technologies.

• **Blockchain:** Blockchain-supported FL applications are being implemented to solve problems, such as centralized processing, data manipulation, and lack of motivation [60]. With blockchain technology, improvement of the security and scalability of FL is also being realized [42]. Singh et al. [61] proposed a framework to use in the IoT healthcare field, taking advantage of the unanimity efficiency improvement of FL and the stability, originality, transparency, distributed, and decentralized features of blockchain. It has been stated that although the study was developed only as a theoretical model, it is planned to focus on delay and storage needs in the future [61]. BLADE-FL, a blockchain-based decentralized FL architecture, was proposed in [62]. The standard FL frameworks work with a single central server. Hence, a malicious client may cause the training to fail. In BLADE-FL, FL and blockchain are integrated into each client for training and mining tasks, respectively. Studies based on blockchain-based FL were overviewed in [63]. It was concluded from the literature that integrating blockchain with FL increases security in training, but is not sufficient to protect privacy.
• **Edge Computing:** Since FL is a collaborative learning and model optimization method, it can be an encouraging factor for edge computing technology that extends computing services closer to the dataset [64]. Poisoning attacks, an attack model using edge computing in FL, were designed by Zhang et al. [65]. Updating the global model parameters by repeating the samples of the clients, increasing the applicability of the attacks by reducing the attack assumptions, and poisoning attack strategies such as label flipping and backdoor were implemented. As a result, it was observed that these attacks effectively risked the global model [65]. Ye et al. [66] proposed the EdgeFed algorithm to optimize the high computational cost encountered on mobile devices during the implementation of FL based on edge computing with the FedAvg strategy. In this algorithm, the process of updating the local model is separated, and the outputs of mobile devices are collected at the edge server to reduce the frequency of global communication [66]. Another study for FL model aggregation of image classification was conducted in the field of vehicular edge computing [67]. The model was selected using the two-dimension contract theory as a distributed framework. As a result of studies involving the MNIST dataset, model aggregation showed better results in accuracy and efficiency than the original FedAvg strategy [67].

## 6 FL-based frameworks and tools

In FL research, we find numerous libraries, many of which are open-source, that act as both frameworks and tools. They assist in training models, ensuring security, facilitating communication, and quickly compiling results. Using these libraries helps reduce repetitive tasks in the code, leading to more streamlined models. With the growing variety of these tools, their capabilities also expand. Therefore, choosing the right tool or framework becomes essential for better model outcomes.

### 6.1 Existing frameworks and tools

Various software tools tailored for Federated Learning (FL) are emerging, each with distinct methods and capabilities. The literature showcases several notable frameworks and tools, including but not limited to: TensorFlow Federated (TFF) [68], PySyft [69], NVIDIA Federated Learning Application Runtime Environment (NVFlare) [70], Federated AI Technology Enabler (FATE) [71], Flower [72], IBM Federated Learning (IBM FL) [73], FedLab [74], FedML [75], Federated Learning Utilities and Tools for Experimentation (FLUTE) [76], Open Federated Learning (OpenFL) [77], SecureBoost [78], Interplanetary File System (IPFS) [79], Fed-BioMed [80], and FederatedScope [81]. Subsequent subsections focus on the unique features of these frequently mentioned frameworks.

**Figure 4:**
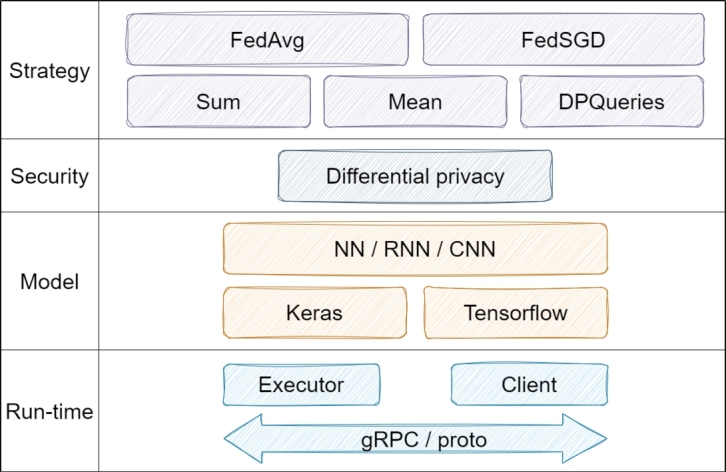
Architecture of TFF [82].
• **PySyft:** Developed by the OpenMined community, PySyft is an open-source framework that prioritizes data privacy and security. It employs methods, such as anonymization, encryption, and differential privacy to partition data and models [69]. Since it performs data anonymization, this framework is used in encrypted privacy-preserving deep learning studies. The differential privacy techniques included in PySyft help prevent attacks against FL models that may be endangered despite being encrypted, by confusing steps that increase the complexity of the training process [84]. During remote data operations, it facilitates learning without direct data copying, maintaining privacy boundaries. Moreover, PySyft is compatible with established ML frameworks like TensorFlow and PyTorch [69]. The image describing the PySyft architecture (Fig. 5) contains the main structure of this framework. In this structure, there are special modules to process horizontally and vertically split data and different techniques used to ensure computational, security and confidentiality. PySyft offers a powerful framework, supporting a wide range of features including secure multi-stakeholder computations, different data partitioning strategies, encryption techniques, and privacy-preserving federative learning.

• **TFF:** TensorFlow Federated (TFF) is an open-source framework introduced by Google, supplying decentralized data to ML [68]. It promotes global model training, leveraging local data without requiring server uploads. The results from individual devices are then centralized for consolidation. Being rooted in TensorFlow, TFF is able for mathematical tasks as well as learning [82]. It offers the FL API and Federated Core (FC) APIs, enhancing the flexibility in developing and applying FL algorithms. While it allows the use of Regression and Neural Network (NN) models in horizontal FL, vertical FL is not supported [83]. Unlike many, TFF is designed for single-host deployments, emphasizing its utility where data privacy and security are paramount. The TFF architecture in Fig. 4 includes a comprehensive framework designed to support federative learning scenarios. This architecture includes optimization strategies such as FedAvg and FedSGD, differential privacy with security features, various model types, and several protocols between an executor and client that enable communication at run-time.

**Figure 5:**
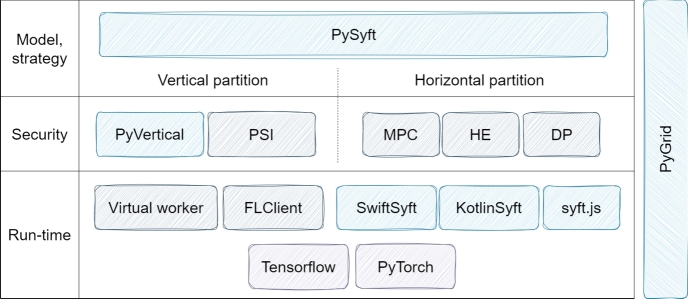
Architecture of PySyft [82].
• **NVFlare:** Designed primarily for collaborative computing, this framework ensures a secure and confidential environment for researchers [85] [70]. It operates using Python. During model training, clients independently train using their datasets and subsequently transmit results to a central server. The server model is updated with this information, and this iterative process persists until the main model convergence [70]. Fig. 6, which shows the NVFlare architecture in detail, includes a wide range of features under the headings of learning algorithms, Federation workflows, FLARE programming API, privacy protection with security management, tools, and FL simulator.

**Figure 6:**
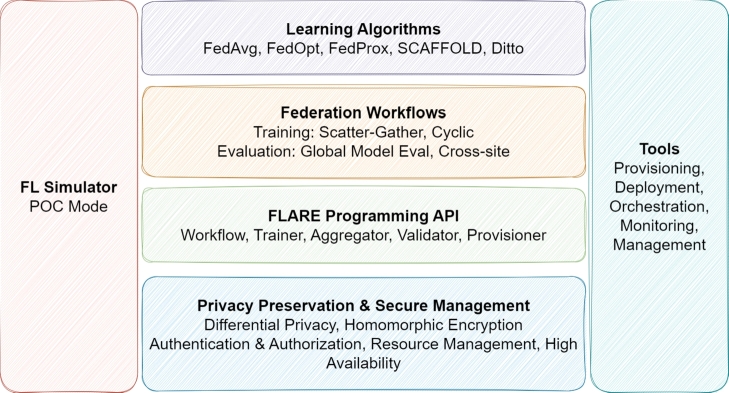
Architecture of NVFlare [86].
• **FATE:** FATE is an algorithm-centric framework focusing on machine privacy protection, with capabilities in both simulation and federated modes [82]. It operates under the assumption of a semi-trusted server, prioritizing combined parameters over private data [71]. Designed for AI and big data industrial applications, FATE finds usage in sectors like finance and healthcare [87] [88]. The framework is versatile, supporting NN, decision trees, regression, and transfer learning [71]. Fig. 7, which shows the FATE framework, reveals in detail a broad structure covering major components such as cloud-based services, workflow management, model distribution, federated ML components, security protocols, computational resources, and storage.

**Figure 7:**
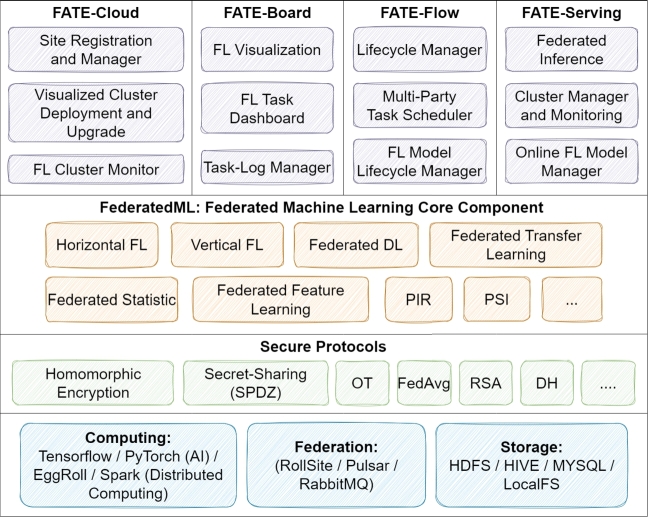
Architecture of FATE [89].
• **Flower:** The Flower FL framework is friendly, easy to use, adaptable to FL models, can be used in similar studies, and can be used to run these studies on lots of heterogeneous devices [72]. Flower has built-in algorithms such as FedAvg, FedProx, QFedAvg, and FedOptim, addressing challenges such as client connectivity issues and adapting to varied network conditions. These core strategies also serve as foundations for deriving algorithms for additional functionalities [72]. The flow in Fig. 8 demonstrates in detail the interaction between the core components of the Flower framework: Strategy, Client Manager and Remote Procedure Call (RPC) Server and RPC Clients, revealing a comprehensive design that shows the workings of Flower's internal structure.

**Figure 8:**
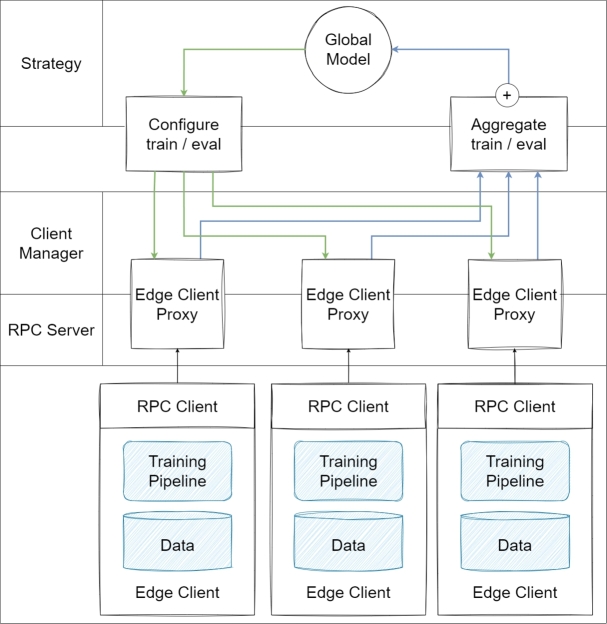
Architecture of Flower [90].
• **IBM FL:** IBM FL, an independent ML library, supports Python and is adaptable for use with decision trees, support vector machines (SVMs), NNs, and RL [73] [82]. Since this FL was developed by IBM company, it is a proprietary framework, distinct from open-source options [82]. FL algorithms are executed by easily configuring all the basic substructures in their infrastructure, and implementation of grouping and aggregation of these algorithms are provided. Its capacity to collate data from various sources enhances its viability for use in institutional areas [73]. Notably, it incorporates FL strategies such as FedAvg and iterative average, predominantly for NN and RL applications [82]. Fig. 9 demonstrates the IBM FL architecture in detail. Data sources of different countries are connected to each country's own Remote Training System (RTS), and it is shown how the model parameters obtained from here are integrated and used within this framework. This flow clearly describes how data sources are integrated and how model updates are coordinated by ensuring secure cross-country collaboration.

**Figure 9:**
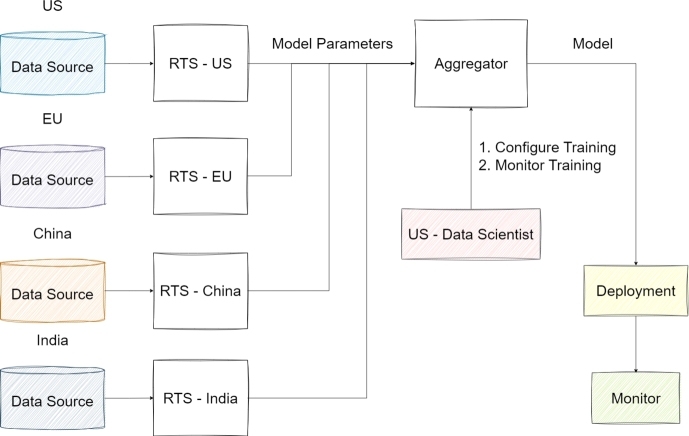
Architecture of IBM FL [91].
• **FedLab:** It is an open-source framework that allows model optimization, data partition, and communication between algorithms and their efficiency can be examined with FL simulations [74]. Because of the flexible structure of the FedLab framework, users can construct their own FL simulation algorithms by modifying the substructures. Three different simulation plans have been added, namely Standalone, Cross-process, and Hierarchical [74]. These modes are used to simulate a single operation by multiple clients with limited possibilities, to process multiple large FL models in the same environment, and to provide communication between local and global servers, respectively [74]. The communication contract definition and FL optimization stages are performed during the model development process. Fig. 10 shows the FedLab architecture with the interaction between the server and the client via the FedLab Communication API. Focusing on FedLab's communication agreement, optimization strategies, and computation steps, the flowchart of the framework is expressed.

**Figure 10:**
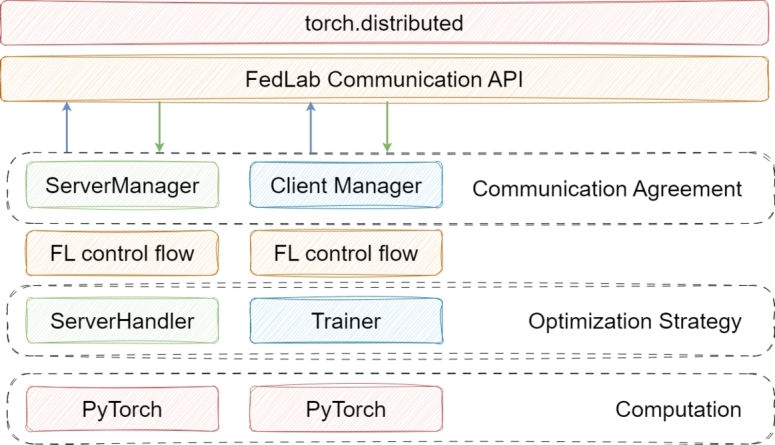
Architecture of FedLab [74].
• **FedML:** FedML is an open-source framework that offers flexibility in training. It supports different computing methods, including distributed computing, on-device training for edge devices, and single-machine simulation [75]. It provides FL training by supporting various algorithmic operations. It consists of two components, FedML-API, which represents the high-level API and enables the implementation of new algorithms in accordance with the client-oriented programming interface, and FedML-core, which represents the low-level API and divides communication and training modules [75]. The design objective behind the low-level APIs is to amplify security, privacy, and resilience. The architecture of the FedML framework consists of FedML Server, FedML-API, and FedML-core structures (as shown in Fig. 11). FedML Server coordinates FL processes, FedML-API performs optimization operations, and FedML-core manages the framework's communication and training operations. Integrated work between and within these structures ensures that the framework provides training in a powerful and efficient manner. These dynamic structures in the architecture of the FedML framework are designed to optimize the learning process and ensure effective communication between different components.

**Figure 11:**
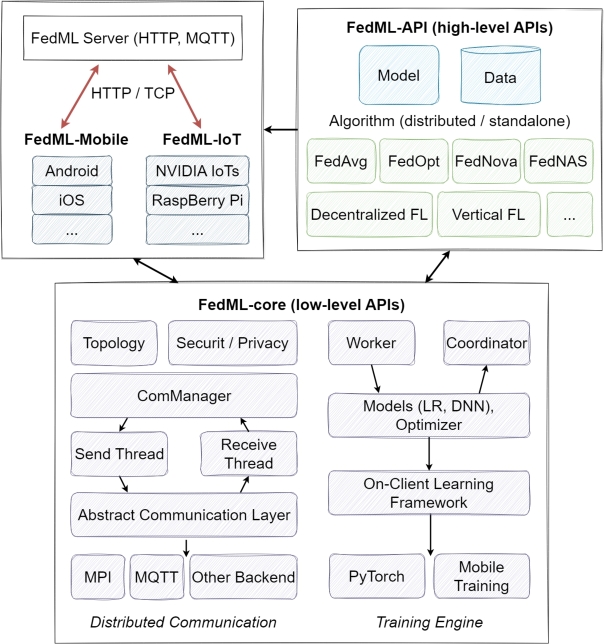
Architecture of FedML [75].
• **FLUTE:** This framework was developed by the Microsoft Research team to ensure enhanced performance in FL research [76]. Tailored for FL simulations, it aims to design a framework that works better in security, optimization, and communication scales [76]. Training procedures can be changed according to the customer's request, and they can accommodate complex algorithms. During the training phase, only gradients are sent to the centralized server, and the client or server can change the gradient values for privacy [76]. FedAvg strategy is used as the basis for federated optimization. Furthermore, it seamlessly integrates with the NVIDIA Collective Communications Library, presenting potential advantages in temporal efficiency and memory consumption [76]. Fig. 12 is a diagram describing the architecture of the FLUTE framework. Here, it is shown how the model on the server is updated, how the data from the clients are integrated, and how the federative approach is applied in the model training process.

**Figure 12:**
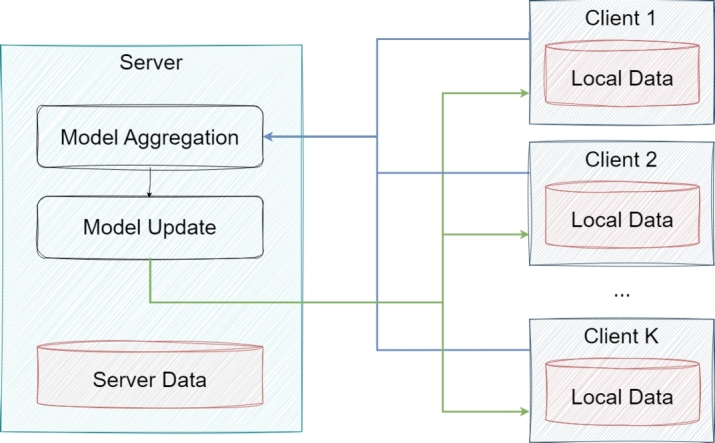
Architecture of FLUTE [92].
• **OpenFL:** OpenFL framework was initially developed for applications in healthcare but has subsequently been updated for broader industrial usage, primarily due to its robust security features [77]. Throughout the model training phase, the model owner can only access model weight updates and measurements. The data used here is retained at the collaborating center [77]. The FL plan and model code can be accessed prior to training with an OpenFL command [77]. It also offers developers the ability to easily work with remote data. However, it doesn't provide differential privacy. Fig. 13 gives the architecture of the OpenFL framework. Here, the flow of model parameters between the collaborator and the aggregator ensures the training process. In addition, the local file system, FL backend, and FL plan executor, which are included in the internal structure of both collaborator and aggregator, constitute the basic infrastructure components of this framework. The workflows within these infrastructures are critical elements that support the general training mechanism of OpenFL.

**Figure 13:**
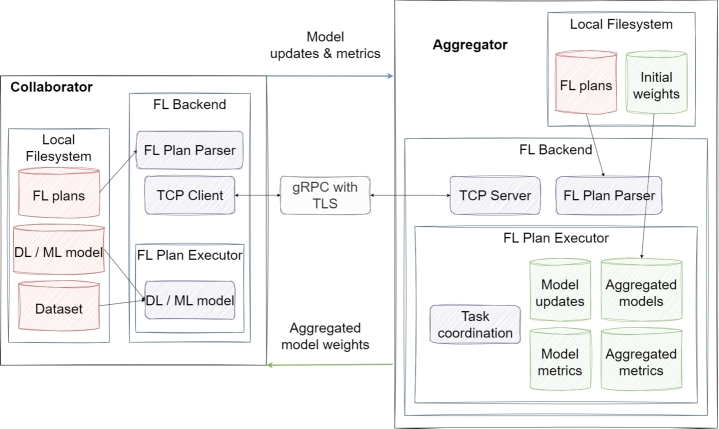
Architecture of OpenFL [77].

### 6.2 Criteria to choose framework

As can be seen from the frames given in the previous section, each frame has its own pros and cons. These features allow users to make different choices according to the FL model they will train. In Table 4, comparisons of these frameworks in terms of various features are given. For example, one of the most essential features that users can benefit from is whether the framework is open source or not. When this matter is examined, it is seen that the sample frameworks given here, except the IBM framework, are open source. As the next feature, when the data sharing permission is investigated, it is stated that many frameworks do not allow vertical FL applications. The ease of use of these frameworks is rated according to the level of availability of manuals such as paper, GitHub, website, and video tutorials. FedML and OpenFL are highly rated for having a large number of manuals. In terms of integration, it has been observed that while TFF can be integrated into the least environment, FedML can be integrated into the most. It is understood from the table that the last three features are taken into account in all frameworks. These can be interpreted as follows. Applications can be implemented with these frameworks in the industrial field. The designs of the models are formed to secure privacy. All of them have a scalable structure.

**Table 4:** Comparison of the properties of the frameworks [93].

	Properties							
Framework	Developer	Open source	Data sharing^1^	Usability^2^	Integration^3^	Industry applications	Safety-focused design	Scalability
TFF	Google	Yes	H	3	T	Yes	Yes	Yes
PySyft	OpenMined	Yes	H & V	3	K, P, T	Yes	Yes	Yes
NVFlare	NVIDIA	Yes	H & V	2	P, T	Yes	Yes	Yes
FATE	WeBank	Yes	H & V	3	K, P, T	Yes	Yes	Yes
Flower	Adap gmbh	Yes	H	1	K, P, T	Yes	Yes	Yes
IBM FL	IBM	No	H	4	K, P, T	Yes	Yes	Yes
FedLab	FedLab	Yes	H	3	K, P, T	Yes	Yes	Yes
FedML	FedML Inc.	Yes	H	5	J, M, P, T	Yes	Yes	Yes
FLUTE	Microsoft	Yes	H	2	P, T	Yes	Yes	Yes
OpenFL	Intel	Yes	H	5	P, T	Yes	Yes	Yes

1 H = Horizontal Federated Learning, V = Vertical Federated Learning.

2 The ease of usage rating is high if the framework has paper, GitHub, website, and video tutorials for its use.

3 J = Jax, K = Keras, M = MXNet, P = PyTorch, T = TensorFlow.

## 7 Opportunities, challenges, and future directions

This section provides an overview of the opportunities, challenges, and future directions of federated learning. Federated learning is a decentralized learning model approach offering improved privacy protection while allowing the collective intelligence of various devices or data resources. However, there are still issues to be addressed, such as communication, system and data heterogeneity, cyber-attacks, etc. Future research and direction in Federated Learning will primarily focus on these challenges.

### 7.1 Opportunities

Federated learning is one of the promising fields in the AI/ML concept. It also offers many opportunities. The primary ones can be summarized below.

• Preserving privacy: Training provided with federated learning is carried out on the local devices of the clients. Only the updated parameters of the model are shared with the server, and the data used in training does not need to be shared [94]. With this method, the confidentiality of the data can be ensured. To provide privacy secure aggregation protocols can be introduced that devise a training framework that can protect the local model updates [95] [96]. These protocols can enable the server to create a combination between the global model's update and each individual local model without learning about any information. This prevents the local model updates from staying concealed from the server which prevents the server from exploiting the updates of any user to infer their private training data [97].
• Improved data security: The data does not leave the device and is processed locally. The information shared with the server is limited to only updated model parameters. Therefore, the server does not have access to any client's own training data.
• Scalability: During the FL process, model training can be carried out in parallel on different devices of the clients. Here, the processed dataset can be divided into users, and its size can be reduced. Additionally, the location of the devices does not matter as long as they have a connection to the server. As a result of the convenience brought by such examples, it can be said that model training with FL is scalable.
• Reduced communication costs: Especially for AI training, the processed datasets must be quite large. Hence, the accuracy of the training increases. In addition, in studies developed collaboratively, a large dataset can be obtained by combining the datasets of different clients. In studies conducted with FL, working with a large amount of data requires less communication cost. Because each client processes its own dataset and shares only the training parameters with the server without sharing the dataset. For this, low bandwidth is sufficient [64].
• Edge computing integration: With Edge Computing (EC), IoT data obtained from devices such as sensors is used in studies [64]. However, this data is directly shared with third parties. Since the data is shared, communication costs are high. Data confidentiality also cannot be ensured. However, it is possible to integrate this architecture with FL. Thus, EC studies can be performed using the opportunities provided by FL.
• Real-time adaptability: As aforementioned, model training is done on local devices. The model parameters obtained as a result of the training are instantly shared with the server. Therefore, clients can directly access the current model. In this way, efficient and fast training processes are carried out in real-time.
• Decreased data bias: In the training performed by different clients on their own devices, data that do not have the same content and type are used. These data can be IID (independent and identically distributed) or non-IID. Thus, data diversity increases. This increases the generalization performance of the updated model and reduces data bias.

### 7.2 Challenges

Research on FL is still in its primary stages. Federated Learning has several challenges. Some of them are discussed below.

• Security and Privacy Challenges: The fundamental premise of Federated Learning is to provide privacy to the local datasets. A secure aggregation algorithm is proposed that can aggregate encrypted local models without decrypting the data in the aggregator [98]. However, a specific local learner's identity can be disclosed. It can be done by analyzing the global model [99]. To prevent this, differentially private federated learning has been proposed [100]. Necessary privacy is provided at the local learner level instead of providing protection to a single data sample.
• Wireless Communication and Settings: The other challenge is wireless communication (widely used in real-world applications) due to limited channel capacity, noise, and interference. The information is quantized before being sent over to the channels. This is done by exchanging model parameters between the local learners and the aggregator. The federated learning paradigm takes off with parameter quantization [101].
• Communication Overloads: In federated learning, it is one of the major challenges. Existing studies tried to solve this issue by applying data compression [4] or by allowing clients to add only relevant output [102], [103].
• System and Data Heterogeneity: In the FL, the system and data heterogeneity in the network along with the non-identically distributed data from the nodes significantly affect the overall model and system performance. An FL model trained on heterogeneous, i.e., non-IID datasets may bias training in the direction that causes heterogeneity of datasets stored locally by clients. As observed in the literature, the problem of data heterogeneity is tried to be overcome by developed strategies used in FL. For example, while FedAvg is more suitable for training with IID datasets, the developed FedProx, FedYogi, Scaffold, etc. strategies were improved to adapt the strategies to non-IID datasets [32], [33], [34].
• Membership Inference Attacks: In this case, the raw data stays in the local device. Even with this step, there are several ways, which can infer the training data used in FL. It is possible to extract the information regarding the training data [104]. It looks for mechanisms that offer a differential privacy guarantee. Some of the defense mechanisms are discussed below.
  **–** Secure Computation: There are two main techniques that fall under this category, i.e., Secure Multiparty Computation (SMC) and Homomorphic Encryption (HE). In SMC, with the inputs provided by the participants only two or more parties comes to an agreement to perform. Also, the output is only revealed exclusively to a subset of participants. In HE, computation is performed on encrypted inputs without decrypting it first [104].
  **–** Differential Privacy: In this scheme, before model aggregation, noise is added to mask a user's contribution. [100].
  **–** Trusted Execution Environment (TEE): It is a secure platform, which runs a process when provided with low computational overhead. This is executed when it is compared with a secure computation technique.
• Data Poisoning Attacks: It is widely recognized as the most prevalent form of attack against ML models. In the context of the FL model, this attack is carried out during the training process by introducing malicious behavior to a subset of participating devices. This results in the compromisation of model accuracy. The adversary has the ability to directly inject poisoned data into the devices or inject poisoned data through other devices [105]. Identifying malicious participants is the defense mechanism against this kind of attack. It is executed in each round of learning before averaging.
• Model Poisoning Attacks: These attacks are similar to data poisoning attacks. In this case, the main objective is to corrupt the local models rather than manipulating the local data. The global model is affected by introducing errors. The attacker carries out a model poisoning attack by compromising certain devices and altering the parameters of the local model. The defense mechanism is almost similar to data poisoning attacks. The most common defense mechanisms are based on rejections. It is mostly based on two factors one is error rate and another one is loss function [106]. To overcome this challenge, Bagdasaryan et al. have developed a model-replacement technique that is accomplished by injecting backdoors [107]. However, the persistence and stealth of this methodology are not good enough [108]. Thus, stealth was tried to be ensured with another methodology developed in [109]. The method was improved with the use of an alternating minimization strategy. Additionally, they noted that their methodology is very vulnerable to Byzantine-resilient aggregation strategies [109]. In the study [108], an attack based on optimizing stealthy and persistent was proposed. This was developed by bypassing defense methods and avoiding forgetting, respectively.
• Backdoor Attacks: Secured Federated Learning provides the devices anonymity during the process of the model update. Backdoor Attacks use the same functionality, a device or a group of devices introduces an adversary in the global model [107]. An attacker can manipulate certain tasks without impacting the overall accuracy of the global model. This can be done by assigning a particular label to a data instance with specific characteristics, which is commonly referred to as a targeted attack.

### 7.3 Future directions

In federated learning, there are many open research directions required to explore, which can address the challenges discussed earlier. They can be briefly explained in the following.

• Wireless Settings: One of the important considerations in paradigm quantization is the robustness of the models present in the quantization error. It also includes communication bandwidth, noise, and interference. Robustness to these channel effects might be a consideration [101].
• System and Data Heterogeneity: FedAvg was introduced as a method to tackle this challenge. However, due to the large differences in the structure of the datasets used for different applications, this method is not efficient in overcoming this. Modifications in the model aggregation methods are addressed to solve this issue [110], [111].
• Incentive Mechanism: FL methods operate under the assumption that devices will collaborate during the learning process as needed, without taking rewards into account. On the contrary, nodes need to be economically compensated for their participation. This reputation-based incentive method, where devices get rewarded according to their model accuracy, data reliability, and contribution, might help get better models [112], [113].
• Federated Learning as a Service: Federated Learning as a cloud service is recommended for collaborating among third parties. This framework allows the third party applications to contribute and collaborate on an ML model. This was developed in a recent work [114]. For any operating environment, the framework claims to be suitable.
• Asynchronous Federated Learning: Current FL aggregation techniques are for devices that has the ability to work in a synchronized manner. However, due to factors such as systems and data heterogeneity along with training and model transfer a synchronized manner is followed. In this manner, the feasibility to scale federated optimization has a chance of decreasing. This can happen in a synchronized manner [115]. Asynchronous federated averaging techniques have the capability to accommodate a larger number of devices, enabling updates to be received at different times in comparison to FedAvg.
• Blockchain in FL: In order to enhance the global model's capability to handle the asynchronous arrival of device parameters, the inclusion of an aggregator is essential. The presence of this component serves as a requirement for the widespread adoption of FL models. Blockchain is an example of a decentralized network. This means devices have the ability to learn collaboratively without using the central aggregator. Federated Learning was proposed by some works in blockchain framework [116].
• Backdoor Attacks: The model remains vulnerable to backdoor attacks by attackers placing backdoor triggers on local models during the training stage. Then, at the prediction stage, attackers triggered by crafted inputs cause misclassification [117]. To overcome this challenge, various backdoor defense methods are being developed [107].
• Poisoning Attacks: It has been determined that FL frameworks have vulnerabilities in active attacks. One of these, the poisoning attack, occurs when attackers damage the global model with local updates prepared by attackers [118]. In addition, data poisoning attacks are sometimes performed by injecting malicious data into the training dataset before the learning stage begins [119].
• Adversarial Example Attacks: In this type of attack, malicious output is produced by adding little disturbances to the input samples [120].

## 8 Conclusion

This survey paper provides a comprehensive overview of Federated Learning (FL), i.e., a distributed machine learning approach, which enables collaborative training of a shared model without sharing raw data. Unlike traditional approaches, FL facilitates collaborative model training without the insecure exchange of raw data, thereby safeguarding critical data privacy and security. FL offers a solution by allowing data to remain decentralized while facilitating model training through collaborative efforts and various strategies of FL. Security concerns related to FL are addressed in the survey, recognizing the importance of safeguarding data and models in distributed learning environments. Furthermore, the survey explores the FL-based applications, revealing their transformative potential across diverse domain(s). Additionally, the survey recognizes both the opportunities and challenges associated with working with FL, underscoring the potential for enhanced machine-learning applications while acknowledging the complexities involved. It is expected that the survey paper will contribute to the advancement and adoption of this innovative approach in the field of federated learning in terms of data privacy, security, and scalability.

## CRediT authorship contribution statement

**Betul Yurdem:** Writing – review & editing, Writing – original draft, Visualization, Validation, Methodology, Investigation, Formal analysis, Conceptualization. **Murat Kuzlu:** Writing – review & editing, Writing – original draft, Supervision, Data curation, Conceptualization. **Mehmet Kemal Gullu:** Writing – review & editing, Writing – original draft, Supervision, Project administration, Formal analysis, Conceptualization. **Ferhat Ozgur Catak:** Writing – review & editing, Writing – original draft, Supervision, Investigation, Formal analysis, Conceptualization. **Maliha Tabassum:** Writing – review & editing, Writing – original draft, Formal analysis, Conceptualization.

## Declaration of Competing Interest

The authors declare that they have no known competing financial interests or personal relationships that could have appeared to influence the work reported in this paper.

## Figures and Tables

**Algorithm 1:**
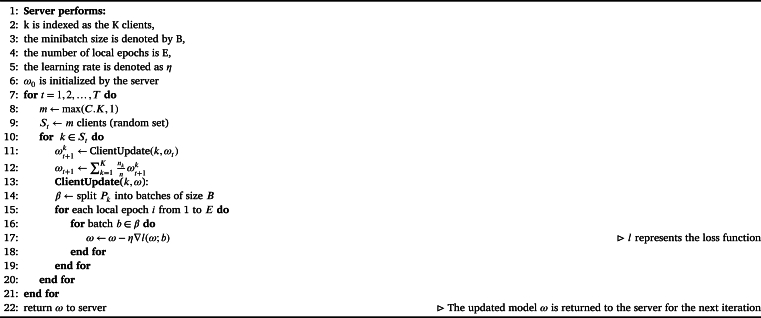
FedAvg [18].

**Algorithm 2:**
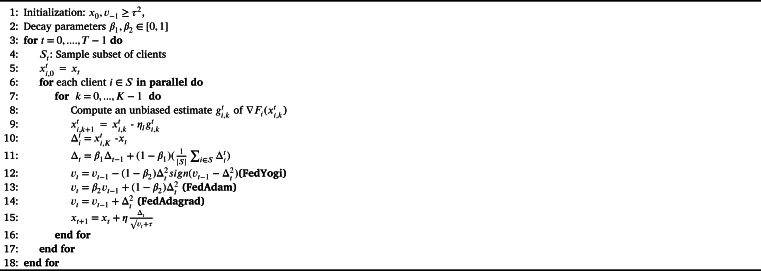
FedYogi, FedAdam and FedAdagrad [121].

**Algorithm 3:**
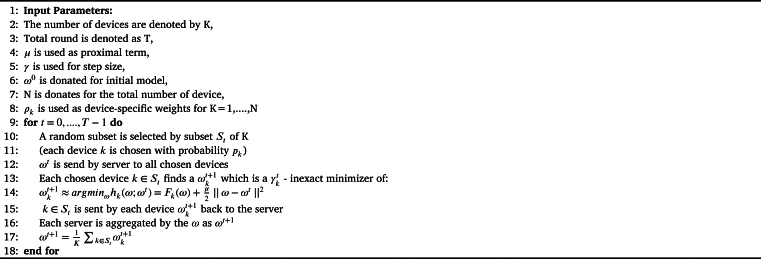
FedProx [122].

**Algorithm 4:**
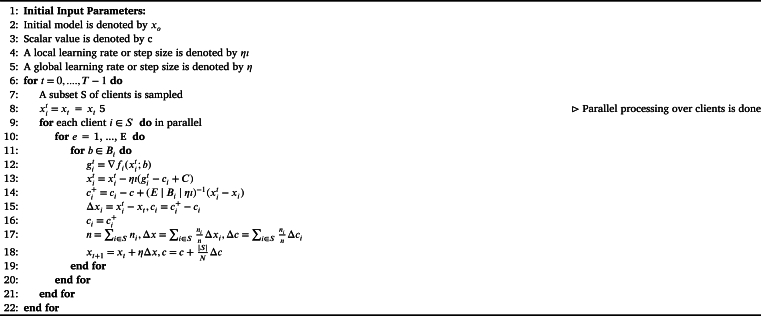
Scaffold [123].

## Data Availability

Not applicable.
